# Acupuncture Topics on Twitter (Currently X) in English and Japanese: Co-occurrence Network Analysis

**DOI:** 10.7759/cureus.54928

**Published:** 2024-02-26

**Authors:** Takumi Kayo, Kazushi Uneda, Masao Suzuki

**Affiliations:** 1 Department of Kampo Medicine, Aizu Medical Center, Fukushima Medical University, Aizuwakamatsu, JPN

**Keywords:** co-occurrence network, japanese, english, twitter (currently x), acupuncture

## Abstract

Background

With the growth of social media, there has been an increase in health-related studies utilizing data obtained from such sites and applications. Although acupuncture is used as a complementary alternative medicine worldwide, there is little research on acupuncture utilizing social media data. This study investigates the topics related to acupuncture on Twitter, currently known as X, in English and Japanese.

Methods

We collected tweets containing the English word “acupuncture” and its Japanese equivalent using Twitter's application programming interface from January 1, 2022 to December 31, 2022. After extracting the top 50 frequently occurring words from the collected tweet texts separately for each language, we conducted a co-occurrence network analysis for those words, in order to evaluate the patterns of their occurrence.

Results

A total of 70,435 English tweets from 41,939 users and 188,671 Japanese tweets from 81,093 users were analyzed after excluding retweets and duplicate tweets. The co-occurrence network analysis revealed that topics related to pain, other complementary and alternative medicines, and acupuncture-related experiences were common in both languages. However, explanatory topics such as needle use, Chinese medicine, and body points were specific to English tweets, while those about beauty care were specific to Japanese tweets.

Conclusion

The number of tweets regarding acupuncture and the number of users who posted them were both higher in Japanese than in English. Some acupuncture topics were common in both languages, while other topics were specific to each language. The findings of this study provide valuable insights into understanding information about acupuncture on social media.

## Introduction

With the growth of social media such as Facebook, Twitter (currently known as X), Instagram, and YouTube, health-related research using data from these media is increasing [1,2]. Social media data have been extensively studied in areas such as mental health, drug side effects, infectious diseases, chronic diseases, and behavioral risk factors [1,2]. Complementary and alternative medicine (CAM) is also frequently discussed in social media. The Pew Research Center reported that 35% of internet users had searched for information related to CAM [3]. Therefore, investigations on CAM using social media data have become an important area of health research [4].

Acupuncture, which involves inserting thin needles into specific body parts, is a popular form of CAM used worldwide. Research on acupuncture has been conducted in over 60 countries, mainly in East Asia and Western countries, with the number of published research studies steadily increasing [5]. However, there has been little research on acupuncture using social media data.

Twitter is a popular social networking site, used for communication with other users by sending and responding to short text messages called “tweets,” which are limited to a maximum of 280 characters. Twitter has become the most common resource on social media for health research [1,2], and it is also used for research on CAM, such as spinal manipulative therapy and medical cannabis use [4].

This study aims to investigate the topics regarding acupuncture on Twitter in both English and Japanese. Twitter hosts tweets in over 150 languages, but these two languages are the most frequently used and account for half of all tweets [6]. Moreover, since acupuncture originated in East Asia and then spread to Western countries, the topics related to acupuncture may differ between the two languages. To the best of our knowledge, this study is the first to investigate the topics regarding acupuncture on Twitter.

## Materials and methods

Data collection

From January 1, 2022 to December 31, 2022 (UTC), we collected publicly available tweets containing “acupuncture” in English and the word which means acupuncture in Japanese. Tweets were collected through Twitter's application programming interface using R (version 4.0.3) and R studio (version 2021.09.0) with R package academictwitteR (version 0.3.1) [7]. This study was exempt from ethical review board approval because it used information in the public domain and did not contain personally identifiable information.

Data cleaning

Retweets were excluded from the collected data. Tweets containing the word “bot” in the usernames or profiles were also excluded. The following processing was applied to each tweet text: removal of emoji, URLs, hashtags, HTML markups, handle tags, punctuation, and other symbols, and numerals; conversion of white spaces to a single space; expansion of English contractions to their long form; conversion of all English words to lowercase; and normalization of Unicode characters with Normalization Form Compatibility Composition. After performing these cleaning steps, we excluded tweets that contain the same text and that do not contain the word “acupuncture” or its equivalent in Japanese. The above data cleaning was performed using the R package textclean (version 0.9.3), dplyr (version 1.1.1), emoji (version 15.0), and stringi (version 1.7.12).

Analysis

We counted the number of tweets per day over the study period for both the total tweets and tweets for analysis after data cleaning. Additionally, we calculated the average number of tweets per day (with minimum and maximum).

We used KH Coder (version 3.Beta.06d) [8], free software for text mining that supports both English and Japanese, to extract frequent words from the text, and then conducted co-occurrence network analyses to evaluate the occurrence patterns of these frequent words. The morphological analyzers for word extraction were Stanford POS tagger for the English tweets and ChaSen for the Japanese tweets. The parts of speech of the extracted words in English were nouns (including proper nouns), adjectives, adverbs, verbs, and foreign words, and those in Japanese were nouns (including “sahen” nouns and proper nouns), adjectives (including “na” adjectives, “nai” adjectives, and non-independent adjectives), adverbs, verbs, and unknown words (words not included in the morphological analysis dictionary).

Words with low analytical value (for example, “do,” “mine,” “about,” etc. in English) were excluded from extraction targets as stop words. For English tweets, words listed in the English stop word list of the natural language processing software spaCy (version 3.1) [9], and the following words “acupuncture,” “acupuncture therapy,” “acupuncture therapies,” “acupuncture treatment,” and “acupuncture treatments” were excluded. Since the words related to acupuncture were included in all tweets and are known to co-occur with other words, they were included in the stop words. For Japanese tweets, the words that consisted of hiragana only were excluded, and as in English tweets, the Japanese words related to acupuncture that were listed as excluded words above were considered as stop words.

We conducted co-occurrence network analyses for the top 50 most frequent words in English and Japanese. Network diagrams were drawn with the extracted words expressed as circular nodes, and their co-occurrence relationships were represented as edges. The strength of the co-occurrence relationship was evaluated using the Jaccard coefficient [10], and the top 60 edges based on the Jaccard coefficient were drawn on the network diagram. Furthermore, the modularity optimization method was applied to detect communities (also called partial networks or subgraphs) in network diagrams where edges are densely interconnected [11]. After drawing the co-occurrence network diagrams, we looked at the raw text of the tweets in order to confirm the topics in which the co-occurring words were mainly used.

## Results

**Figure 1:**
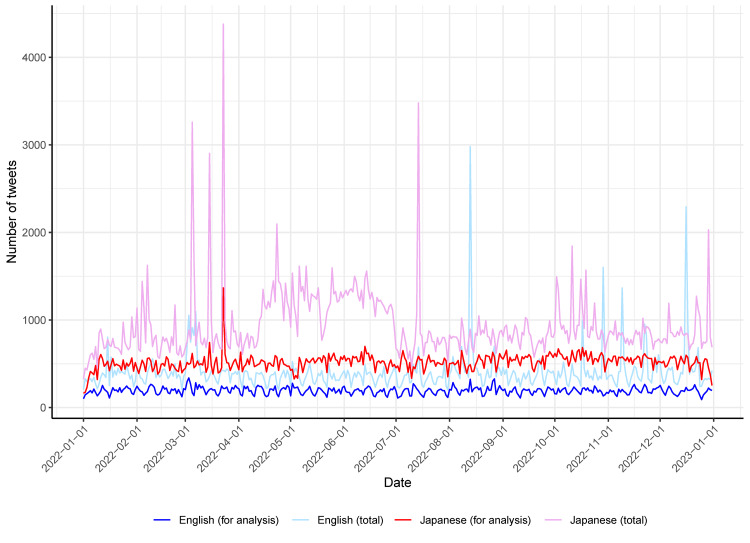
Number of tweets per day

During the study period, a total of 147,890 English-language tweets from 81,560 users were collected. Out of these, 70,435 tweets from 41,939 users were included in the analysis after removing retweets, duplicates, and tweets that did not contain the word “acupuncture.” In Japanese, a total of 342,094 tweets from 135,239 users were collected, out of which 188,671 tweets from 81,093 users were included in the analysis. The average number of English-language tweets per day was 405.2 (minimum 204; maximum 2,980) for all tweets and 193.0 (minimum 91; maximum 339) for analyzed tweets. In Japanese, the average number of Japanese-language tweets per day was 937.2 (minimum 325; maximum 4,379) for all tweets and 516.9 (minimum 164; maximum 1,368) for analyzed tweets. Figure 1 shows the number of tweets per day over the study period.

**Figure 2:**
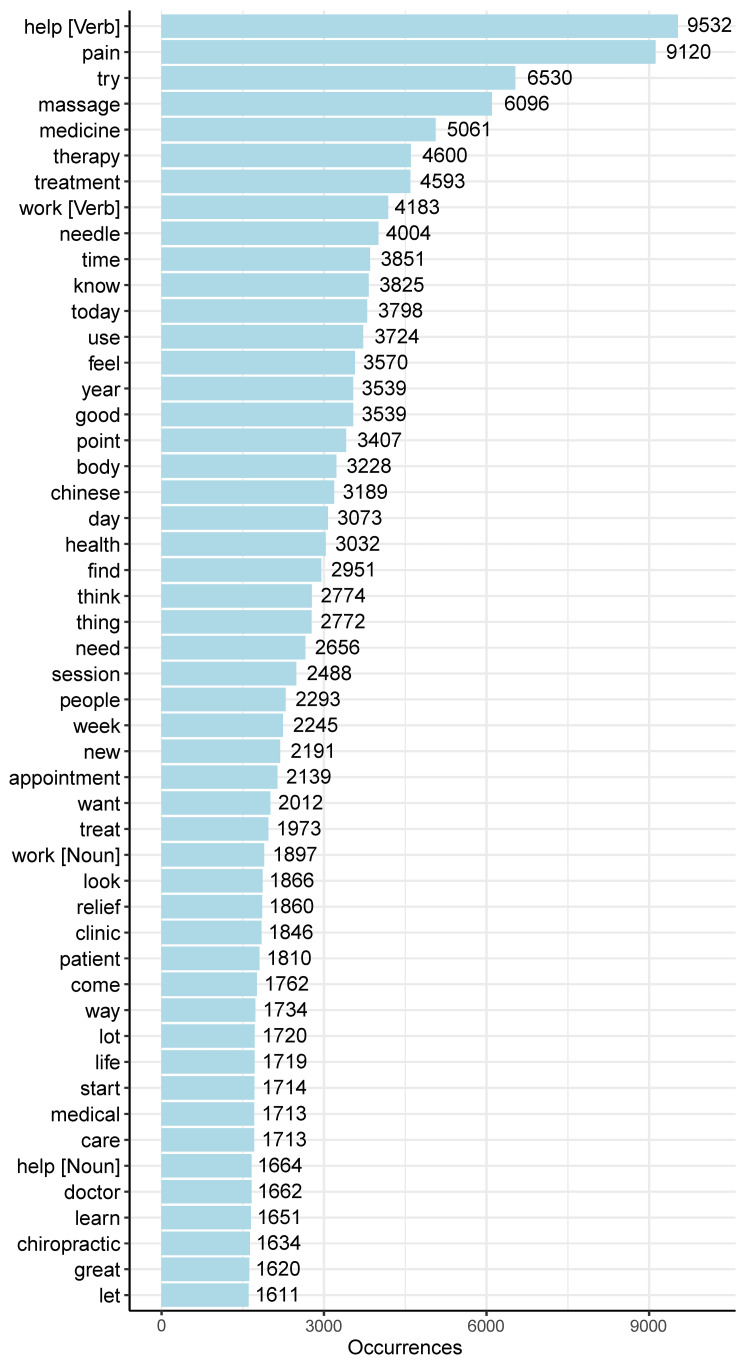
Top 50 most frequent words in English tweets

**Figure 3:**
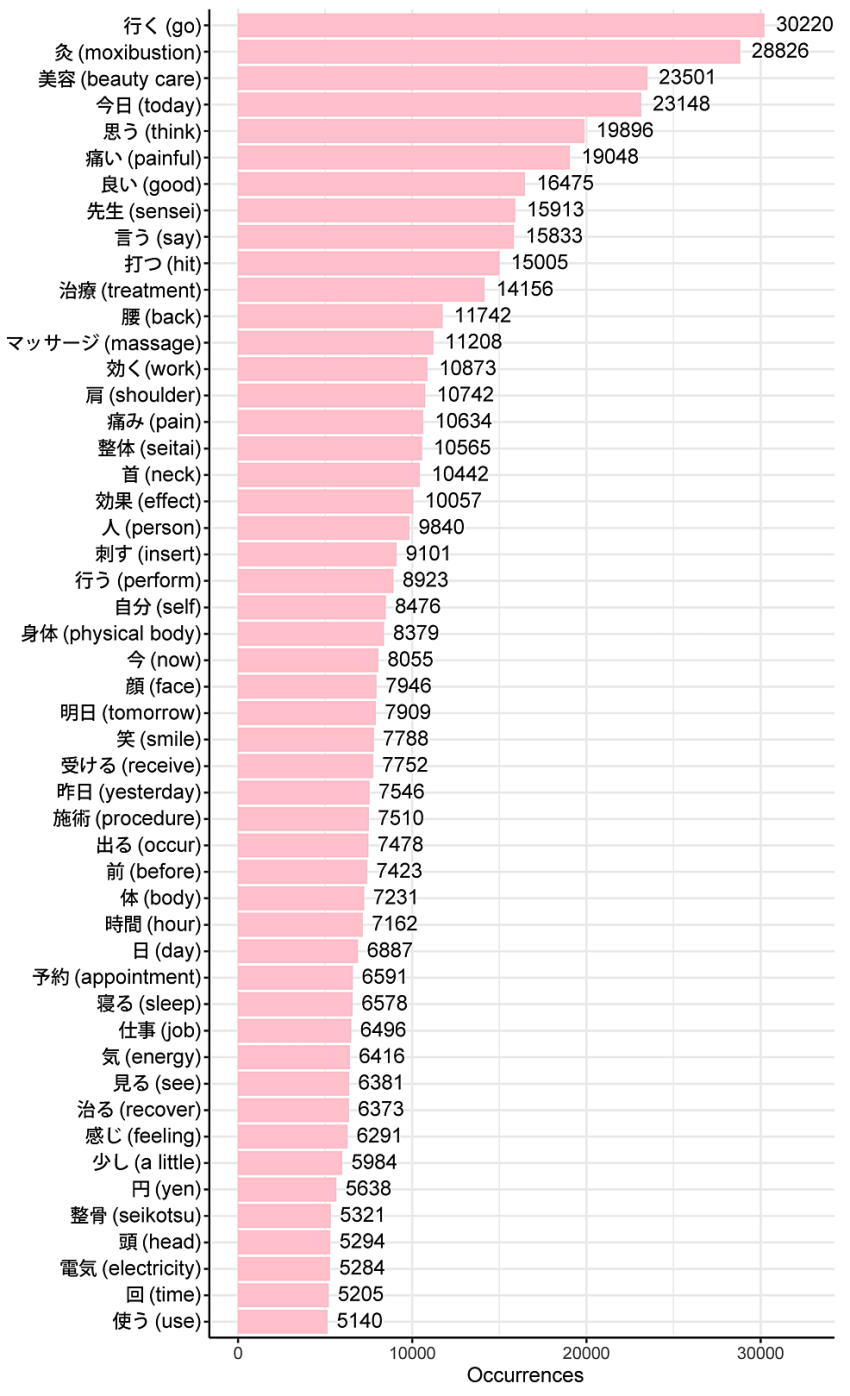
Top 50 most frequent words in Japanese tweets (and corresponding English translations)

After performing morphological analysis on the tweet texts, 770,247 words from 50,351 different words were extracted in English, and 2,308,382 words from 59,123 different words were extracted in Japanese. Figures 2, 3 display the top 50 most frequently used words in English and Japanese tweets, respectively.

**Figure 4:**
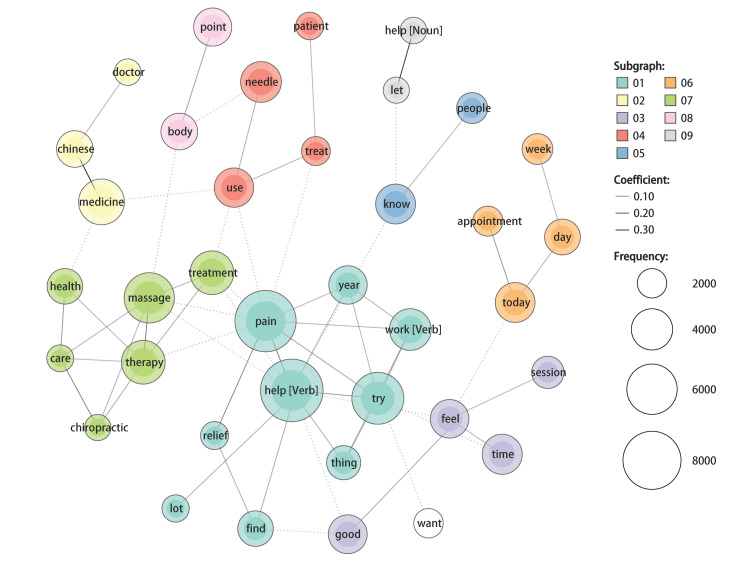
Co-occurrence network of words in English tweets In this network diagram, the extracted words are expressed as circular nodes, and their co-occurrence relationships are represented as edges. The size of the nodes increases according to the frequency of word usage, and the edges are thicker based on the strength of the relationship evaluated using the Jaccard coefficient [10]. The node colors are assigned based on the detected communities, which are referred to as subgraphs in the figure.

Co-occurrence network analysis was performed on both the English and Japanese tweet texts. In the English tweets, nine communities were detected. The top three frequently occurring words in English tweets, namely “help,” “pain,” and “try,” formed a community with the eighth-ranked word “work” and others. These words were primarily used in the context of acupuncture for pain. The fourth-ranked word “massage” formed a community with the sixth-ranked “therapy,” the seventh-ranked “treatment,” and others such as “chiropractic.” These words were primarily used in topics where acupuncture and other CAMs were mentioned simultaneously. The fifth-ranked word “medicine” formed a community with “Chinese” and “doctor,” which were primarily used in topics related to acupuncture and traditional Chinese medicine or traditional Chinese doctors. The ninth-ranked word “needle” formed a community with “use” and “treat,” and these words were primarily used in topics of acupuncture as a treatment using needles. The 10th-ranked word “time” formed a community with “feel” and “good,” and these words were primarily used in topics about feelings and impressions before and/or after receiving acupuncture (e.g., feeling good after a session). The community including “today” and “appointment” was primarily used in topics about scheduling to receive acupuncture on a specific date, and the community consisting of “point” and “body” was primarily used in topics of specific sites of the body and acupuncture (Figure 4).

**Figure 5:**
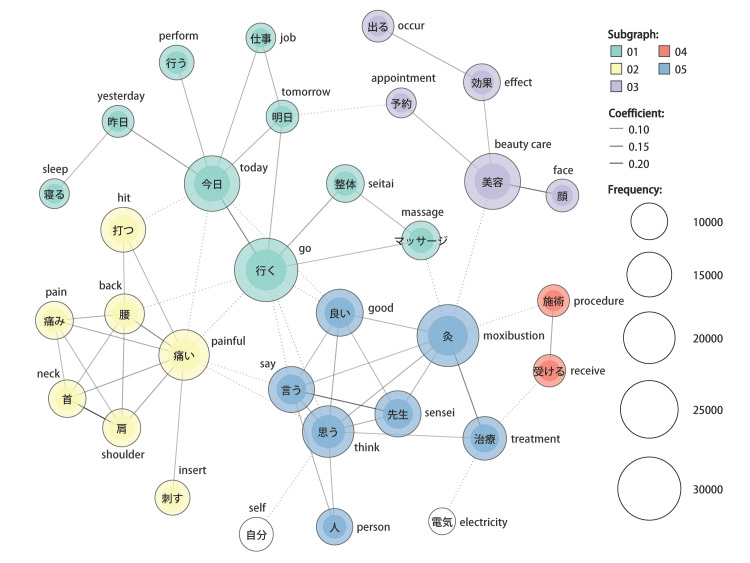
Co-occurrence network of words in Japanese tweets In this network diagram, the extracted words are expressed as circular nodes, and their co-occurrence relationships are represented as edges. The size of the nodes increases according to the frequency of word usage, and the edges are thicker based on the strength of the relationship evaluated using the Jaccard coefficient [10]. The node colors are assigned based on the detected communities, which are referred to as subgraphs in the figure. The corresponding English translations are appended to each Japanese word.

In the Japanese tweets, five communities were detected. The most frequently occurring word in Japanese tweets was the word meaning “go” in Japanese, which formed a community with the fourth-ranked word “today” in Japanese (the same hereinafter) and others such as “massage” and “seitai (a kind of traditional Japanese manipulative therapy).” These words were used primarily in topics about scheduling to receive acupuncture on specific dates and receiving other CAMs at the same time. The second-ranked word “moxibustion” formed a community with the fifth-ranked word “think” as well as the seventh-ranked word “good,” the eighth-ranked word “sensei (a Japanese honorific title used to refer to professionals in their fields, such as teachers, doctors, acupuncturists, artists, and lawyers),” the ninth-ranked “say,” and others. These words were primarily used in topics about impressions of acupuncture and moxibustion, and conversations with acupuncturists. The third-ranked word “beauty care” formed a community with words such as “effect” and “face,” and these words were primarily used in topics about acupuncture for beauty purposes. The sixth-ranked word “painful” formed a community with the 10th-ranked word “hit (utsu)” and others such as “back” and “shoulder.” In Japanese, the verb “hit (utsu)” is used to mean “to insert (a needle)” in the context of acupuncture. These words were used primarily in topics about acupuncture for bodily pain. The remaining of the five communities was the one formed by the words “procedure” and “receive” (Figure 5).

## Discussion

We conducted co-occurrence network analyses of the words in tweet texts to investigate the topics related to acupuncture expressed in English and Japanese tweets throughout the year 2022. The number of collected tweets and the number of users who posted them were higher in Japanese compared to English, and we identified that some topics were common in both languages, whereas others were distinct to each language. To the best of our knowledge, this is the first study on acupuncture using Twitter data. In terms of acupuncture research using social media data, there have only been investigations into the accuracy of information on YouTube videos regarding acupuncture for COVID-19 and breech presentation [12,13]. Therefore, we could not find any previous studies directly comparable to our findings.

We observed more tweets related to acupuncture and users posting such tweets in Japanese than in English. This difference may be influenced by the prevalence of acupuncture, including the number of acupuncturists and acupuncture users, in each language region. The number of licensed acupuncturists was reported to be 38,000 in the United States as of 2018 [14], and 5,000 in Australia as of 2020 [15]. In contrast, Japan has 186,000 licensed acupuncturists as of 2021 [16]. The annual utilization rates of acupuncture were reported as 1.7% in the United States in 2012 [17], 1.6% in the United Kingdom in 2004 [18], and 2.4% in Canada in 2010 [19]. In contrast, the reported rate was 5.7% in Japan in 2022 [20]. These data suggested that Japanese people may be more interested in acupuncture because of their ease of access. Furthermore, the higher prevalence of acupuncture in Japan compared to English-speaking countries may make Japanese people more knowledgeable about acupuncture and do not need explanations about it. In English, there were explanatory acupuncture topics, such as needle use, body points, and Chinese medicine, whereas such topics were not observed in Japanese tweets.

The topic of pain was commonly observed in both the English and Japanese tweets. In English, the word “pain” was the second most frequent, and in Japanese, the words “painful” and “pain,” when combined, ranked second (totaling 29,682 words). Previous studies have reported that the most common reason for acupuncture use in the United States, the United Kingdom, and Japan is painful conditions, such as musculoskeletal problems [17,21,22]. As a matter of fact, acupuncture is known to be effective for musculoskeletal pain [23]. Therefore, the study results suggest that pain is one of the significant topics to Twitter users worldwide, and they seem to have a firm understanding of acupuncture as a treatment for pain.

The word “massage” appeared in both languages as a CAM-related term. Furthermore, “massage” co-occurred with terms for other manual therapies such as “chiropractic” in English and “seitai” in Japanese. These manual therapies are widely used for musculoskeletal problems in both English-speaking countries and Japan [24-26]. Therefore, acupuncture may be mentioned simultaneously with these manual therapies on Twitter by individuals interested in treatment for musculoskeletal problems, with a similar motivation for using these CAMs. On the other hand, the word “moxibustion” appeared exclusively in the Japanese tweets. In Japan, even when people receive treatment with acupuncture only, they tend to call it “acupuncture and moxibustion (shinkyu).” The reason why the word “moxibustion” was so common in Japanese tweets may be because phrases such as “acupuncture and moxibustion clinic (shinkyu-in)” and “acupuncture and moxibustion doctor (shinkyu-shi)” were tweeted. Additionally, in English-speaking countries, moxibustion is often considered an additional treatment performed by acupuncturists [27], whereas in Japan it is performed by moxibustionists, who hold a distinct professional qualification separate from acupuncturists [16]. The higher occurrence of “moxibustion” in Japanese tweets may be also because moxibustion is distinguished from acupuncture in Japan.

We observed tweets about acupuncture plans and impressions in both languages, indicating that Twitter users share their personal experiences related to acupuncture. Online sharing of personal experiences is a common empowerment process among individuals with chronic illnesses [28]. Sharing their acupuncture experiences on Twitter may help them enhance their skill to self-manage their illnesses. Notably, many Japanese users tweeted about their conversations with acupuncturists. A previous study has reported that, compared to the United States, Japanese patients value their healthcare providers’ opinions more highly with regard to medical decisions [29]. Therefore, conversations with acupuncturists may be considered to be significant experiences for Japanese Twitter users, leading to a higher frequency of tweeting about those experiences.

Topics related to beauty care were characteristic of the Japanese tweets. Acupuncture for beauty care is a relatively new field in Japan, and related information in Japanese on the internet has increased rapidly over the past 10 years, which is reportedly three to four times more common than in English [30]. Therefore, our findings may reflect the recent increased interest in this area by Japanese acupuncture users and acupuncture-related businesses.

Strengths and limitations

The strengths of this study include an extensive investigation into acupuncture topics on Twitter by targeting two languages that constitute the majority of Twitter users. Additionally, data collection over one year allowed analyses of topics in tweets without being influenced by seasonal variations.

However, the current study has several limitations. Firstly, the analysis was limited to public tweets; therefore, it did not include surveys of protected tweets. Secondly, due to the anonymity of Twitter, demographic characteristics such as age, gender, and nationality of the posters are unknown, making it difficult to accurately identify the target population of the study. Thirdly, although it was anticipated that there would be differences in topics related to acupuncture among acupuncture users, non-users, and providers, the collected tweets were mixed, and it was not possible to distinguish between these groups. Lastly, the generalizability of the present study’s findings to other social media platforms is not guaranteed.

## Conclusions

The number of tweets regarding acupuncture and the number of users who posted them were higher in Japanese compared to English. Tweets in both languages had common topics, such as pain, other CAMs, and acupuncture-related experiences. However, the English tweets had characteristic topics such as needle usage, Chinese medicine, and specific body points, while the topic of beauty care was distinctive in the Japanese tweets. The findings of this study will provide valuable knowledge for understanding information about acupuncture on social media, which is increasingly influential.
